# The Diagnostic Accuracy of Bone Marrow Biopsy Versus PET/CT Scan in Identifying Bone Marrow Involvement in Diffuse Large B Cell Lymphoma Patients at a Cancer Hospital

**DOI:** 10.7759/cureus.34901

**Published:** 2023-02-12

**Authors:** Hiba Asif, Rabia Zubair, Imran A Siddiqui, Muhammad Tariq Mahmood, Ahsan Jamil, Ammarah Tahir

**Affiliations:** 1 Pathology and Laboratory Medicine, Shaukat Khanum Memorial Cancer Hospital and Research Centre, Lahore, PAK; 2 Internal Medicine, Shaukat Khanum Memorial Cancer Hospital and Research Centre, Lahore, PAK

**Keywords:** pet/ct, bone marrow biopsy, diagnostic test accuracy, diffuse large b cell lymphoma (dlbcl), bone marrow involvement

## Abstract

Background

It is of great importance to assess bone marrow involvement (BMI) in diffuse large B cell lymphoma (DLBCL) for staging, prognostic, and therapeutic purposes. The gold standard method used for the identification of bone marrow involvement is bone marrow biopsy (BMB), but it has certain drawbacks. In recent years, positron emission tomography/computed tomography (PET/CT) has become a highly effective method in the diagnosis and staging of lymphoma.

Objective

The objective of this study is to estimate the diagnostic accuracy of PET/CT in identifying bone marrow involvement in DLBCL patients in a cancer care hospital in Lahore, using BMB as a reference standard.

Methods

This descriptive cross-sectional study was conducted at the Department of Pathology of Shaukat Khanum Memorial Cancer Hospital and Research Centre (SKMCH&RC) from January 1, 2013, to December 31, 2018. A retrospective data of 146 patients fulfilling the inclusion and exclusion criteria was retrieved from the hospital information system (HIS). The inclusion criteria include patients aged 18-80 years, of either gender, and with a confirmed diagnosis of DLBCL on tissue biopsy. The exclusion criteria include patients who had started chemotherapy or radiotherapy for DLBCL or were using granulocyte colony-stimulating factor (G-CSF) prior to their PET/CT scan. All patients underwent PET/CT and BMB, and the diagnostic accuracy of PET/CT was calculated, with BMB taken as the reference standard.

Results

The mean age of cases was 52.73 ± 16.27 years. There were 95 (65.1%) male and 51 (34.9%) female cases, with a high male-to-female ratio. In the present study, 32.19% of cases had bone marrow involvement on BMB, and 34.2% of cases had bone marrow involvement on PET/CT. The sensitivity, specificity, positive predictive value (PPV), negative predictive value (NPV), and overall diagnostic accuracy of PET/CT were found to be 93.61%, 93.93%, 88%, 96.88%, and 93.84%, respectively.

Conclusion

It is concluded that PET/CT scan has good sensitivity, specificity, positive predictive value, negative predictive value, and diagnostic accuracy. So, it is suggested to choose this non-invasive technique because the presence of a disease in extra-medullary space can also be detected and the evaluation of bone marrow in the whole body can be performed. PET/CT scan is an effective imaging modality in the detection of bone marrow involvement in DLBCL patients, and its relative advantages over bone marrow biopsy might conclude this to be a preferred technique.

## Introduction

Non-Hodgkin's lymphoma (NHL) is the most frequent hematological cancer with approximately 3%-4% of all cancer diagnoses and 6% mortality all around the globe [1,2]. Non-Hodgkin's lymphoma includes a wide variety of different neoplastic diseases; the most frequently occurring NHL is diffuse large B cell lymphoma (DLBCL) (nearly 30%-40% of adult cases) [1,3,4], and DLBCL is characterized by a comparatively elevated number of extra-nodal proliferation [3,5]. Bone marrow involvement (BMI) by DLBCL is seen in about 27% of cases, evaluated by bone marrow biopsy (BMB) at the time of diagnosis [6]. Bone marrow involvement (BMI) affects lymphoma staging, management, and outcomes [7]. The detection of bone marrow involvement is preeminent in the staging, treatment, and prognosis of newly identified DLBCL [7-9].

One of the most common methods used for the detection of BMI by the underlying lymphoma is bone marrow biopsy (BMB) [10]. Although BMB is considered a gold standard and a safe procedure for the evaluation of BMI, it has certain limitations and risks [11,12]. It is an invasive and painful procedure and might not yield adequate material for pathologists to interpret [10]. It may also miss patchy or focal bone marrow lymphoma outside the iliac crest area [10,12]. Unfavorable outcomes such as bleeding, infection, allergy, and failure of procedure have also been reported in around 0.12% of patients [11]. In recent years, positron emission tomography/computed tomography (PET/CT) has become an effective method in the diagnosis and staging of lymphoma [13]. In recent retrospective studies, the diagnostic and prognostic value of PET/CT to determine BMI remains controversial [14,15].

The current study aims to find the diagnostic accuracy of PET/CT in identifying BMI in DLBCL patients in the local Pakistani population, taking bone marrow biopsy as a reference standard. Local data regarding this topic is scarce in this part of the world especially Pakistan; hence, our study will add valuable insight.

## Materials and methods

This descriptive cross-sectional study was conducted at the Department of Pathology of Shaukat Khanum Memorial Cancer Hospital and Research Centre (SKMCH&RC), Lahore, from January 1, 2013, to December 31, 2018.

A retrospective data of 146 patients fulfilling the inclusion and exclusion criteria was retrieved from the hospital information system (HIS). The inclusion criteria include patients aged 18-80 years, of either gender, and with a confirmed diagnosis of DLBCL on tissue biopsy. The exclusion criteria include patients who had started chemotherapy or radiotherapy for DLBCL or were using granulocyte colony-stimulating factor (G-CSF) prior to their PET/CT scan. Bone marrow biopsy specimens were sent to the pathology laboratory, and PET/CT scan was directed to the Department of Nuclear Medicine for reporting.

PET/CT imaging and reporting

**Figure 1:**
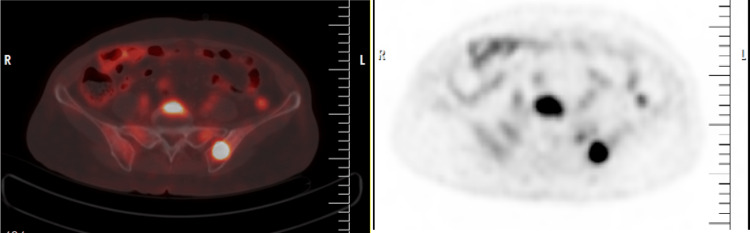
PET/CT PET/CT showing FDG-avid lesion with osseous involvement in a patient with diffuse large B cell lymphoma PET/CT, positron emission tomography/computed tomography; FDG, fluorodeoxyglucose

PET/CT was done as whole-body scans (from the base of the skull to the mid-thigh) before which the patient was advised to fast overnight or for at least 4-6 hours before injection in order to avoid carbohydrates in the meal. Patients underwent blood glucose tests before administering 18F-fluorodeoxyglucose (18F-FDG), which is a radio-labelled biological compound, to ensure suitably low levels of glucose, ideally <150 mg/dl for optimal image quality; otherwise, high glucose levels can interfere in fluorodeoxyglucose (FDG) uptake and can affect the accuracy of the result. The standard 18F-FDG dose is 10-15 mCi and is given intravenously. To allow the proper distribution and uptake of the radiotracer (18F-FDG), the patient was allowed to rest quietly for 60 minutes in a shielded room, in order to avoid artifacts and to minimize FDG uptake in the muscle and brown fat. Imaging was acquired using Gemini TF 16 scanner (Philips, Amsterdam, the Netherlands), with scanning from the base of the skull to the mid-thigh. Coronal, sagittal, and transversal PET/CT projections were reconstructed and analyzed using the software. PET/CT scan was considered positive if the value of standardized uptake volume (SUV) maximum of FDG increases in bone marrow as compared to the liver. Standardized uptake volume (SUV) is the ratio of radioactivity activity concentration (kBq/ml) measured by the PET scanner within a region of interest (ROI) to the decay-corrected amount of injected radio-labelled FDG (kBq) per kilogram weight of the patient. The findings of PET/CT scan were reported by an experienced radiologist and nuclear medicine physician (Figure 1).

Bone marrow biopsy and reporting

**Figure 2:**
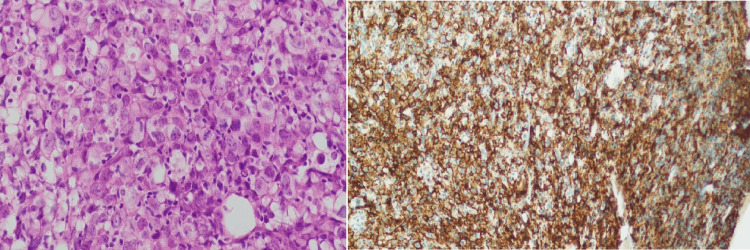
Bone marrow trephine biopsy Bone marrow trephine biopsy showing that diffuse infiltration with large lymphoma cells with a moderate amount of cytoplasm and CD20 is strongly positive in these large cells CD20: cluster of differentiation 20

Unilateral bone marrow biopsy was performed from the posterior superior iliac spine to get an adequate (2-3 cm) sample of bone marrow trephine and placed immediately in Bouin's solution (formaldehyde, trinitrophenol, and acetic acid) for fixation and sent to the Department of Histopathology. After giving appropriate time for fixation (fixation time varies depending upon the fixative solution used), the specimen is decalcified in a decalcification solution (hydrochloric acid and formic acid) and then placed in an automatic tissue processor machine (Leica or Peloris, Leica Biosystems, Wetzlar, Germany) for dehydration (using the increasing strength of alcohol to dehydrate the tissue), clearing (to remove alcohol and be replaced by wax by immersing the tissue in xylene), and impregnation with wax. Thereafter, the bone marrow biopsy specimen is embedded in paraffin wax blocks, and 4 μm-thick sections are cut with rotary microtomes. These sections are then stained with hematoxylin-eosin and subsequently evaluated morphologically by a consultant hematopathologist. Bone marrow involvement on bone marrow biopsy was considered if there was a morphological evidence of lymphoma involvement in the sections examined supported by immunohistochemical stains (cluster of differentiation 20 {CD20}, CD3, CD10, and Ki67) (Figure 2).

All data was collected by the researcher himself. The collected data was entered and analyzed using Statistical Package for Social Sciences (SPSS) version 24 (IBM SPSS Statistics, Armonk, NY). Mean ± SD was used for quantitative data (such as age). Frequency and percentage were calculated for qualitative data such as bone marrow involvement (BMI) on BMB and PET/CT. A 2 × 2 table was made for findings of PET/CT and BMB to calculate the sensitivity, specificity, positive predictive value (PPV), negative predictive value (NPV), and diagnostic accuracy of PET/CT, taking BMB as reference standard.

## Results

A total of 146 cases were included in the study. The mean age of cases was 52.73 ± 16.27 years with an age range of 18-80 years. There were 95 (65.1%) male and 51 (34.9%) female cases with a high male-to-female ratio. There were 47 (32.19%) cases who had bone marrow involvement on BMB and 50 (34.2%) cases who had bone marrow involvement on PET/CT as shown in Table 1.

**Table 1:** Baseline characteristics of study participants (N = 146) Continuous data is given as mean ± standard deviation while categorical, discrete data as number and percentage N, number; BMI, bone marrow involvement; BMB, bone marrow biopsy; PET/CT, positron emission tomography/computed tomography

Baseline characteristics		
Age (years)		52.73 ± 16.27
Gender	Male	95 (65.07)
	Female	51 (34.93)
BMB involvement	Yes	47 (32.19)
	No	99 (67.81)
PET/CT involvement	Yes	50 (34.25)
	No	96 (65.75)

There were 44 cases who had bone marrow involvement on BMB and PET/CT (true positive), and 93 cases were negative on both modalities (true negative). There were six false positives (BMI on PET/CT but not on BMB) and three false negatives (BMI on BMB but not on PET/CT). The sensitivity, specificity, PPV, NPV, and overall diagnostic accuracy of PET/CT were found to be 93.61%, 93.93%, 88%, 96.88%, and 93.84% respectively, as shown in Table 2.

**Table 2:** Diagnostic accuracy of bone marrow involvement on PET/CT, taking BMB as reference standard (N = 146) PET/CT, positron emission tomography/computed tomography; BMB, bone marrow biopsy; PPV, positive predictive value; NPV, negative predictive value; N, number

Bone marrow involvement		On BMB			Sensitivity	Specificity	PPV	NPV	Diagnostic accuracy
		Yes	No	Total	93.61%	93.93%	88.0%	96.88%	93.84%
On PET/CT	Yes	44	6	50					
	No	3	93	96					
	Total	47	99	146					

## Discussion

Bone marrow evaluation at the time of diagnosis is of great importance in the management of patients with lymphoma, as its involvement may indicate an advanced disease [16]. Bone marrow biopsy (BMB) is still the standard method used for lymphoma staging worldwide [6]. But due to certain limitations of BMB, PET/CT has become an effective tool in detecting bone marrow involvement by lymphoma due to its ability to detect both nodal and extra-nodal disease sites and help in staging and predicting prognosis [16]. Bone marrow involvement detected by either method upgrades staging, increases International Prognostic Index (IPI), and is associated with poor outcomes in DLBCL patients [8]. Various chemotherapeutic medicines, steroids, and the targeted immunotherapy medication rituximab are typically used to treat DLBCL in the form of a combination such as rituximab, cyclophosphamide, doxorubicin hydrochloride, vincristine, and prednisolone (R-CHOP) with or without radiation therapy depending upon the stage of the DLBCL. A majority of patients respond to 6-8 cycles of R-CHOP and are cured, while 10%-15% show primary refractory disease, and another 20%-30% relapse [17].

In a study conducted by Teagle et al. [12], it was concluded that FDG PET/CT has 100% sensitivity and specificity for detecting bone marrow involvement in DLBCL, keeping bone marrow biopsy as the reference standard. In a recent systematic review by Almaimani et al. [10], including 41 studies, the total number of patients with DLBCL was 2336; among the included studies with patients' data of DLBCL for PET/CT, the sensitivity, specificity, PPV, and NPV were 77.40%, 91.65%, 63.60%, and 97.00%, respectively, and the sensitivity of BMB in finding out BMI was 47.00%, specificity was 100%, PPV was 100%, and NPV was 80.00%. In another recent study conducted by Xiao-Xue and coworkers [18], it was found that the sensitivity, specificity, and accuracy of PET/CT to detect BMI in aggressive B cell lymphoma (DLBCL and Burkitt lymphoma) were 80.0%, 90.0%, and 88.1%, respectively. A study conducted by El Karak et al. [9] showed that PET was more sensitive than BMB (92.3% versus 38.5%) in detecting bone marrow involvement by DLBCL, and specificity was 100% for both tests.

In another study conducted by Soydal et al. [15], the sensitivity, specificity, accuracy, PPV, and NPV of 18F-FDG PET/CT in the detection of BMI were calculated as 100%, 96%, 96%, 75%, and 100%, respectively. A retrospective study conducted in 2014 by Adams et al. [14] demonstrated that PET/CT detected bone marrow involvement in 43.6% of cases and BMB in 20.5% of 78 cases, of whom 11 were also detected by FDG PET/CT, resulting in a patient‐based sensitivity of 68.8% for FDG PET/CT. Similarly, another study performed by Cortés-Romera et al. [13] showed that PET/CT scan can identify more bone marrow involvement in DLBCL and Hodgkin's lymphoma as compared to bone marrow biopsy with sensitivity, specificity, accuracy, and positive and negative predictive values of 95%, 86%, 87%, and 54% and 99%, respectively. One study from Pakistan by Shaikh et al. [19] confers that PET/CT scan can precisely identify bone marrow infiltration in DLBCL patients and has good diagnostic accuracy.

In the present study, there were 32.19% of cases who had bone marrow involvement on BMB, and 34.25% of cases had bone marrow involvement on PET/CT scan, and we found that PET/CT scan was able to identify bone marrow involvement in most cases. The results from the other studies support the findings of the current study that PET/CT is better than BMB for the detection of bone marrow involvement in DLBCL.

The limitation of our study is that it includes only adult age group and lacks pediatric age group data. It is a single-center study and might not be reflective of state population. More studies need to be done to make PET/CT scan a standard diagnostic modality for the accurate staging of DLBCL patients.

## Conclusions

It is concluded that the diagnostic accuracy of PET/CT scan has good sensitivity, specificity, positive predictive value, negative predictive value, and diagnostic accuracy. So, it is suggested to choose this non-invasive technique because the presence of a disease in extra-medullary space can also be detected and the evaluation of bone marrow in the whole body can be performed.

PET/CT scan is an effective imaging modality in the detection of bone marrow involvement in DLBCL patients, and its relative advantages over bone marrow biopsy might conclude this to be a preferred technique.

## References

[1] Thandra KC, Barsouk A, Saginala K, Padala SA, Barsouk A, Rawla P (2021). Epidemiology of non-Hodgkin’s lymphoma. Med Sci (Basel).

[2] Siegel RL, Miller KD, Fuchs HE, Jemal A (2022). Cancer statistics, 2022. CA Cancer J Clin.

[3] de Leval L, Jaffe ES (2020). Lymphoma classification. Cancer J.

[4] Susanibar-Adaniya S, Barta SK (2021). 2021 update on diffuse large B cell lymphoma: a review of current data and potential applications on risk stratification and management. Am J Hematol.

[5] Chen Y, Zhou M, Liu J, Huang G (2018). Prognostic value of bone marrow FDG uptake pattern of PET/CT in newly diagnosed diffuse large B-cell lymphoma. J Cancer.

[6] Campbell J, Seymour JF, Matthews J, Wolf M, Stone J, Juneja S (2006). The prognostic impact of bone marrow involvement in patients with diffuse large cell lymphoma varies according to the degree of infiltration and presence of discordant marrow involvement. Eur J Haematol.

[7] Özpolat HT, Yilmaz E, Goksoy HS, Özpolat S, Dogan Ö, Unal SN, Nalcaci M (2018). Detection of bone marrow involvement with FDG PET/CT in patients with newly diagnosed lymphoma. Blood Res.

[8] Nakajima Y, Fujisawa S, Nigauri C (2016). Identification of bone marrow involvement of FL: comparison of PET-CT and bone marrow biopsy. Blood.

[9] El Karak F, Bou-Orm IR, Ghosn M (2017). PET/CT scanner and bone marrow biopsy in detection of bone marrow involvement in diffuse large B-cell lymphoma. PLoS One.

[10] Almaimani J, Tsoumpas C, Feltbower R, Polycarpou I (2022). FDG PET/CT versus bone marrow biopsy for diagnosis of bone marrow involvement in non-Hodgkin lymphoma: a systematic review. Appl Sci.

[11] Muzahir S, Mian M, Munir I (2012). Clinical utility of ¹⁸F FDG-PET/CT in the detection of bone marrow disease in Hodgkin's lymphoma. Br J Radiol.

[12] Teagle AR, Barton H, Charles-Edwards E, Dizdarevic S, Chevassut T (2017). Use of FDG PET/CT in identification of bone marrow involvement in diffuse large B cell lymphoma and follicular lymphoma: comparison with iliac crest bone marrow biopsy. Acta Radiol.

[13] Cortés-Romera M, Sabaté-Llobera A, Mercadal-Vilchez S, Climent-Esteller F, Serrano-Maestro A, Gámez-Cenzano C, González-Barca E (2014). Bone marrow evaluation in initial staging of lymphoma: 18F-FDG PET/CT versus bone marrow biopsy. Clin Nucl Med.

[14] Adams HJ, Kwee TC, Fijnheer R, Dubois SV, Nievelstein RA, de Klerk JM (2014). Bone marrow 18F-fluoro-2-deoxy-D-glucose positron emission tomography/computed tomography cannot replace bone marrow biopsy in diffuse large B-cell lymphoma. Am J Hematol.

[15] Soydal C, Koksoy EB, Yasar A, Turgal E, Erdogan BD, Akbulut H, Kucuk NO (2016). Prognostic importance of bone marrow uptake on baseline (18)f-FDG positron emission tomography in diffuse large B cell lymphoma. Cancer Biother Radiopharm.

[16] Elamir Y, Elazab M, Owis AS, Elsayed HF (2020). PET/CT and bone marrow biopsy (BMB) in evaluating bone marrow in lymphoma. Egypt J Radiol Nucl Med.

[17] Chaganti S, Illidge T, Barrington S (2016). Guidelines for the management of diffuse large B-cell lymphoma. Br J Haematol.

[18] Xiao-Xue W, Xinyue H, Lijun Z (2020). Whole body FDG-PET/CT for the assessment of bone marrow infiltration in patients with newly diagnosed lymphoma. Med Clin (Barc).

[19] Shaikh MU, Shakeel D, Hassan M, Afzal N, Ali N, Adil S (2022). Diagnostic accuracy of positron emission tomography-computed tomography (PET-CT scan) in detecting bone marrow involvement in patients with diffuse large B cell lymphoma. Ann Pak Inst Med Sci.

